# AI performance in emergency medicine fellowship examination: comparative analysis of ChatGPT-4o, Gemini 2.0, Claude 3.5, and DeepSeek R1 models

**DOI:** 10.55730/1300-0144.6083

**Published:** 2025-06-12

**Authors:** İshak ŞAN, Medine AKKAN ÖZ, Mehmet YORTANLI, Murat GENÇ, Bensu BULUT, Ayşenur GÜR, Hüseyin MUTLU, Ramiz YAZICI, Mustafa Önder GÖNEN

**Affiliations:** 1 Department of Emergency Medicine, Health Science University, Bilkent City Hospital, Ankara, Turkiye; 2 Department of Emergency Medicine, Gülhane Training and Research Hospital, Ankara, Turkiye; 3 Department of Emergency Medicine, Konya Numune Hospital, Konya, Turkiye; 4 Department of Emergency Medicine, Ankara Training and Research Hospital, Ankara, Turkiye; 5 Department of Emergency Medicine, Health Science University, Gülhane Training and Research Hospital, Ankara, Turkiye; 6 Department of Emergency Medicine, Etimesgut State Hospital, Ankara, Turkiye; 7 Department of Emergency Medicine, Faculty of Medicine, Aksaray University, Aksaray, Turkiye; 8 Department of Emergency Medicine, Health Science University, Kanuni Sultan Suleyman Training and Research Hospital, İstanbul, Turkiye; 9 Department of Emergency Medicine, Meram State Hospital, Konya, Turkiye

**Keywords:** Emergency medicine, artificial intelligence, language, fellowship

## Abstract

**Background/aim:**

This study evaluated the accuracy rates and response consistency of four different large language models (ChatGPT-4o, Gemini 2.0, Claude 3.5, and DeepSeek R1) in answering questions from the Emergency Medicine Fellowship Examination (YDUS), which was administered for the first time in Türkiye.

**Materials and methods:**

In this observational study, 60 multiple-choice questions from the Emergency Medicine YDUS administered on 15 December 2024, were classified as knowledge-based (n = 26), visual content (n = 2), and case-based (n = 32). Each question was presented three times to the four large language models. The models’ accuracy rates were evaluated according to overall accuracy, strict accuracy, and ideal accuracy criteria. Response consistency was measured using Fleiss’ Kappa test.

**Results:**

The ChatGPT-4o model was the most successful in terms of overall accuracy (90.0%), while DeepSeek R1 showed the lowest performance (76.7%). Claude 3.5 (83.3%) and Gemini 2.0 (80.0%) demonstrated moderate success. When analyzed by category, ChatGPT-4o achieved the highest success with 92.3% accuracy in knowledge-based questions and 90.6% in case-based questions. In terms of response consistency, the Claude 3.5 model (Fleiss’ Kappa = 0.68) showed the highest consistency, while Gemini 2.0 (Fleiss’ Kappa = 0.49) showed the lowest. Inconsistent hallucinations were more frequent in the Gemini 2.0 and DeepSeek R1 models, whereas persistent hallucinations were less common in the ChatGPT-4o and Claude 3.5 models.

**Conclusion:**

Large language models can achieve high accuracy rates for knowledge and clinical reasoning questions in emergency medicine but show differences in terms of response consistency and hallucination tendency. While these models have significant potential for use in medical education and as clinical decision support systems (CDSS), they need further development to provide reliable, up-to-date, and accurate information.

## 1. Introduction

Rapid developments in artificial intelligence technology are causing significant changes in healthcare and medical education [1]. In recent years especially, large language models (LLMs) based on Chat Generative Pretrained Transformer (ChatGPT) have shown great promise in areas such as medical assessment, diagnostic support, and educational continuity [2–4]. These language models, which use artificial neural networks with billions of parameters, are capable of generating comprehensive response and human-like texts [3–5]. One way to evaluate the medical knowledge and reasoning of these models is to assess their performance on standardized treatment tests. In recent years, many studies have been conducted on LLMs such as ChatGPT-4o (OpenAI, San Francisco, CA, USA), Gemini 2.0 (Google DeepMind, London, UK), Claude 3.5 (Anthropic, San Francisco, CA, USA), and DeepSeek R1 (DeepSeek AI, Beijing, China) on various medical exams such as the American Medical Licensing Examination (USMLE), the Japanese Medical Licensing Examination, and the American Heart Association (AHA) certification exams [3–8]. These studies have shown that the treatment knowledge and reasoning abilities of LLMs vary significantly, and that the performance of some can now approach that of human experts [4–8]. By its very nature, emergency medicine is a specialty that requires quick decision-making skills, extensive medical knowledge, and the ability to analyze complex clinical scenarios [1,2]. As the number of patients that are being seen in emergency departments and the prevalence of diagnostic uncertainty increases, so does the need for tools that can assist clinicians in their decision-making processes [1,2]. In this context, artificial intelligence applications such as large language models are considered to potentially support emergency physicians in their clinical decision-making processes. However, for these technologies to be considered safe for use in clinical settings, their medical knowledge and reasoning skills need to be fully assessed and understood.

Fellowship exams are comprehensive examinations that assess both the theoretical knowledge and the clinical reasoning skills of candidates [9,10]. ChatGPT has been reported to be successful in the fellowship examination (YDUS) and in other examinations taken by fellows [6–11]. The aim of this study was to compare the accuracy and consistency of the responses of four different large language models (ChatGPT-4o, Gemini 2.0, Claude 3.5, and DeepSeek R1) to the Emergency Medicine YDUS questions. The study aims to evaluate the overall accuracy rates of these models, their performance on knowledge-based, visual-content, and case-based questions, and their response consistency. The types of hallucinations produced by the models will also be analyzed.

## 2. Materials and methods

The response accuracy and consistency of the ChatGPT-4o, Gemini 2.0, Claude 3.5, and DeepSeek R1 models in answering emergency medicine YDUS questions were investigated in this observational study. In Türkiye, the YDUS prepared by the Student Selection and Placement Center (ÖSYM) has been administered in specialties other than emergency medicine since 2010, and the Emergency Medicine YDUS was administered for the first time on 15 December 2024^1^. The Emergency Medicine YDUS consists of 60, five-option multiple-choice questions and is prepared by ÖSYM in Turkish. In preparing the exam questions, ÖSYM used the basic source textbooks for Emergency Medicine (Tintinalli’s Emergency Medicine: A Comprehensive Study Guide, 9th Edition and Rosen’s Emergency Medicine: Concepts and Clinical Practice, 10th Edition) as reference. In our study, we used four large language models (LLMs). The first was ChatGPT-4o. ChatGPT-4 Omni (ChatGPT-4o) is currently known to have the highest level of medical domain knowledge among its peers [12]. As of September 2024, this model was considered the most advanced in terms of medical domain knowledge among its peers[13,14]. The second model used was Gemini 2.0, selected because it had significantly outperformed others in generating reliable and accurate references for medical research entries [6]. The third LLM, Claude 3.5, was used because its accuracy and quality in the healthcare domain is similar to that of physicians [7]. DeepSeek R1 was used because it is reported to be a promising artificial intelligence (AI) tool to facilitate diagnosis of diseases and conditions [8].

The multiple-choice Emergency Medicine YDUS questions were obtained from the official website (https://ais.osym.gov.tr/bireyselgiriş/yandalsoruları) between 15 and 25 December 2024. The 60 questions in the Emergency Medicine YDUS were independently evaluated by authors R.Y. and H.M. and classified into three categories: knowledge-based, visual-content, and case-based questions. The questions that were classified differently by these two authors were reviewed and finalized by the third author, İ.Ş.. The examination used in the study consisted of a total of 60 questions, of which 32 (53.3%) were case-based, 26 (43.3%) were knowledge-based, and two (3.3%) involved visual-content. This classification reflects the structure of the assessment tool rather than the outcomes of the study. The Emergency Medicine YDUS questions were entered by B.B. on the same computer on three separate days between 1 and 30 January, using the ChatGPT-4o, Gemini 2.0, Claude 3.5, and DeepSeek R1 models. Three responses were generated per question by each model. This approach was similar to studies that ask LLMs the same question three times each to ensure consistency and response stability [5–11]. The accuracy rates of the models were assessed using overall accuracy, strict accuracy and ideal accuracy.

Overall accuracy: All three responses had to be correct for the model to be considered accurate.

Strict accuracy: If at least two out of three answers were correct, the model was considered accurate.

Ideal accuracy: If at least one of the three answers was correct, the model was considered accurate.

In addition, the responses given by each model were classified as correct or incorrect, and inconsistencies between responses were categorized as either “inconsistent hallucination” and “persistent hallucination”.

Inconsistent hallucination: A situation where a model gives different answers to the same multiple-choice question in different sessions without producing any data.Persistent hallucination: A situation where a model repeatedly gives the same wrong answer to the same multiple-choice question in every session without producing any data.The ÖSYM Emergency Medicine YDUS questions, their correct answers, and the responses of the ChatGPT-4o, Gemini 2.0, Claude 3.5, and DeepSeek R1 models were recorded in a separate Microsoft Excel 2023 document (version 16.73; Microsoft Corp., Redmond, WA, USA). Because only AI models were evaluated in this study and no human or animal subjects were involved, ethics committee approval was not required.

### 2.1. Statistical analysis

Statistical analyses were performed using SPSS 27.0 (IBM Corp., Armonk, NY, USA). Categorical variables were expressed as numbers and percentages, and analyzed using the chi-square test or Fisher’s exact test. Cochran’s Q test was used to compare the performance of the models. Statistical differences between models were assessed using McNemar’s test for pairwise comparisons. Fleiss’ Kappa test was used to measure the consistency between model responses across three separate sessions. The kappa test was classified as moderate agreement for 0.21–0.40, moderate agreement for 0.41–0.60, and significant agreement for 0.61–0.80. [15] Results

In this study, the accuracy rates and response consistency of four different large language models (ChatGPT-4o, Gemini 2.0, Claude 3.5, and DeepSeek R1) in answering YDUS questions were evaluated. The comparative performances of these models in terms of overall accuracy, strict accuracy, and ideal accuracy are shown in the Table. While the overall accuracy in answering Emergency Medicine YDUS questions was highest in the ChatGPT-4o model (90.0%), the DeepSeek R1 model had the lowest rate (76.7%). The performance of the Gemini 2.0 (80.0%) and Claude 3.5 (83.3%) models was moderate. In terms of strict accuracy, ChatGPT-4o had the highest rate (95.0%), while the DeepSeek R1 model had the lowest (85.0%). In the ideal accuracy analysis, the performance of all models was similar, and ChatGPT-4o achieved the highest accuracy with a rate of 96.7%.

However, there was no significant difference in accuracy among the four models (p > 0.05) (Table).

**Figure 1:**
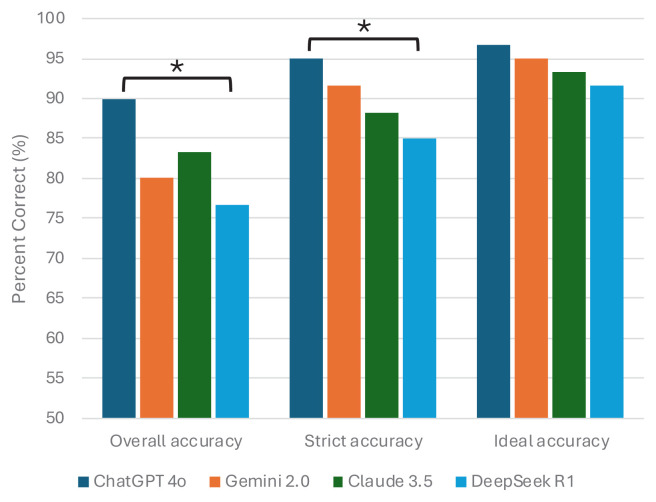
Comparison of overall performance among large language models. The asterisk (*) denotes statistically significant differences between models based on McNemar’s test. Specifically, significant differences were found between ChatGPT-4o and DeepSeek R1 for overall accuracy (p = 0.021) and strict accuracy (p = 0.031).

When the models were compared pairwise, the difference between ChatGPT-4o and DeepSeek R1 was found to be significant in terms of overall accuracy (p = 0.021). Similarly, the difference between these two models was also statistically significant in terms of strict accuracy (p = 0.031) (Table; Figure 1).

**Figure 2:**
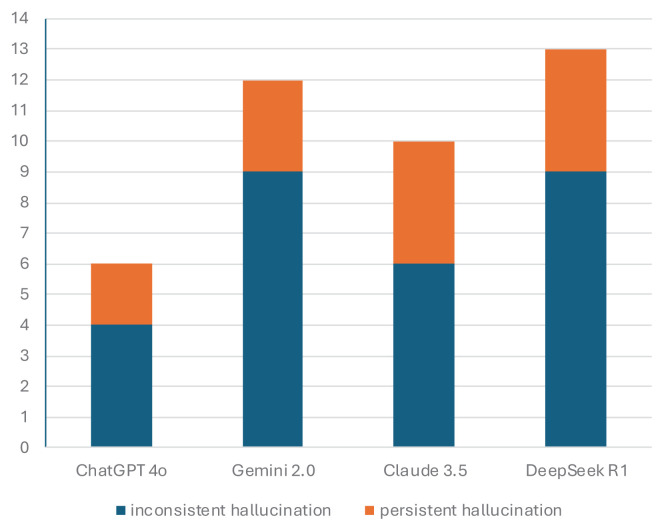
Performance percentages of models based on question types.

When analyzed by category, DeepSeek R1 gave incorrect answers to all questions with visual content (n = 2). For knowledge-based questions (n = 26), ChatGPT-4o had the highest accuracy rate (92.3%), while Gemini 2.0 had the lowest (76.9%). For case-based questions (n = 32), ChatGPT-4o was the best with 90.6% accuracy, while DeepSeek R1 was the worst with 78.1% accuracy (Table; Figure 2).

**Figure 3:**
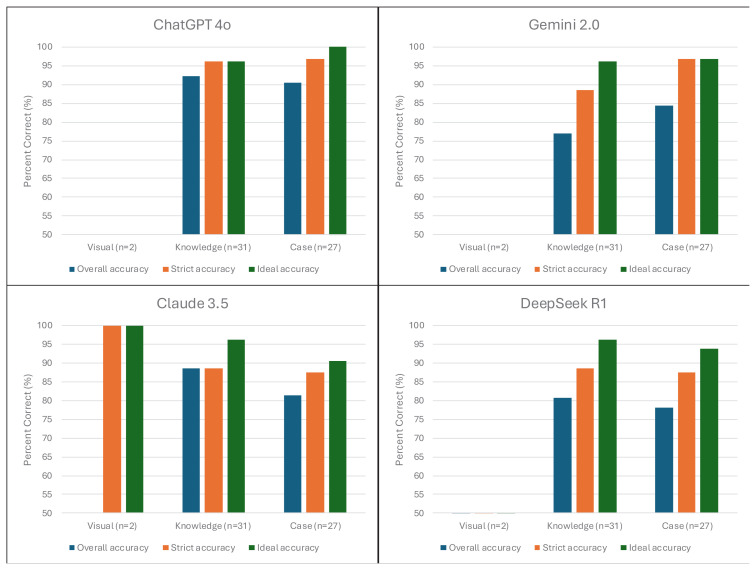
Distribution of large language models based on response accuracy categories. This figure presents the distribution of response accuracy for each question across the four large language models (ChatGPT-4o, Gemini 2.0, Claude 3.5, and DeepSeek R1). For every question, each model generated three responses, which were categorized as follows: strict accuracy (3) – all three responses were correct; general accuracy (2) – at least two responses were correct; ideal accuracy (1) – at least one response was correct; incorrect (0) – all three responses were incorrect.

When the models were asked the same questions three times in order to evaluate consistency, the Claude 3.5 model (Fleiss’ Kappa = 0.68; 95% CI 0.53–0.82; p < 0.001) showed the highest consistency rate. ChatGPT-4o (Fleiss’ Kappa = 0.61; 95% CI 0.47–0.76; p < 0.001) and DeepSeek R1 (Fleiss’ Kappa = 0.62; 95% CI 0.47–0.77; p < 0.001) showed similarly good consistency, whereas Gemini 2.0 (Fleiss’ Kappa = 0.49; 95% CI 0.35–0.64; p < 0.001) showed moderate consistency. These results suggest that while the responses of the Claude 3.5 and ChatGPT-4o models were more consistent, the Gemini 2.0 model showed significantly more variable responses (Figure 3).

**Figure 4:**
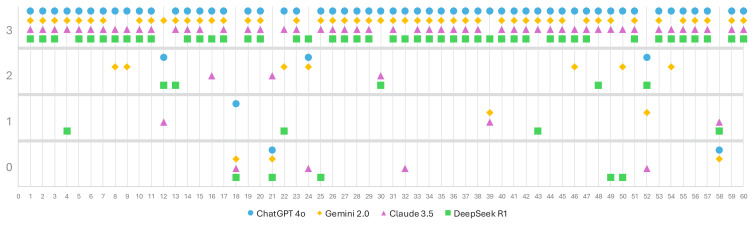
Numerical distribution of response hallucinations among models.

The study also analyzed whether the models gave inconsistent or incorrect answers to the same questions across sessions. Inconsistent hallucinations were particularly common in Gemini 2.0 and DeepSeek R1. Persistent hallucinations were less common in ChatGPT-4o and Claude 3.5, but more common in DeepSeek R1 and Gemini 2.0 (Figure 4). These findings suggest that when assessing the reliability of AI models’ responses, response stability should be considered in addition to their overall accuracy.

## 3. Discussion

In recent years, the evaluation of medical knowledge and clinical reasoning skills of artificial intelligence systems has become an increasingly important research area [7,14]. Our study is the first to evaluate the performance of ChatGPT-4o, Gemini 2.0, Claude 3.5, and DeepSeek R1 in the first Emergency Medicine Specialist Exam implemented in Türkiye. The findings of our study show that ChatGPT-4o has the highest overall accuracy rate (90.0%), followed by Claude 3.5 (83.3%), Gemini 2.0 (80.0%), and DeepSeek R1 (76.7%). Meanwhile, in terms of consistency of responses, the Claude 3.5 model had the highest consistency (Fleiss’ Kappa = 0.68). These results provide important data for the evaluation of medical knowledge and reasoning skills of artificial intelligence models. In a study conducted by Bicknell et al., it was reported that the ChatGPT-4o model achieved an accuracy rate of 90.4% for USMLE disciplines and clinical skills and 92.7% for diagnostic accuracy [16]. Sismanoglu et al. found that the ChatGPT-4.0 and Gemini advanced models were successful in the clinical questions of the Turkish Dentistry Specialization Exam (DUS) with an accuracy rate of 83.3% and 65.0%, respectively [17]. Sabri et al. reported that in answering the American Academy of Periodontology clinical examination questions, ChatGPT-4 had a high accuracy rate of 79.57%, which was similar to Gemini (72.86%) and ChatGPT-3.5 (64.93%) [18]. Abbas et al. found that the ChatGPT-4 model had an accuracy rate of 100% in knowledge-based questions of the Board of Medical Examiners (NBME), followed by Claude with 84.7% [19]. Similarly, Lee et al. reported that ChatGPT-4 and Bing Chat (75.6% and 70.7%, respectively) performed better than ChatGPT-3.5 and Bard (56.9% and 51.2%, respectively) on knowledge-based questions in a study of Korean Emergency Medicine Board exam questions [1]. Similar to these studies, in our study, ChatGPT-4o (92.3%) had the highest accuracy rate in knowledge-based questions, and Gemini 2.0 (76.9%) had the lowest. For case-based clinical questions, ChatGPT-4o ranked first with an accuracy rate of 90.6%, while DeepSeek R1 had the lowest performance with an accuracy rate of 78.1%. These findings show that AI systems are successful in evaluating isolated medical knowledge, but there is still room for improvement in analyzing complex clinical scenarios, and that clinical decision-making processes should be further optimized in future versions of AI systems [20].

A study by Mihalache et al. conducted in ophthalmology highlighted that the ChatGPT-4 model had a success rate of 81.1% on USMLE-style questions, but its visual interpretation ability was limited [21]. Azadi et al. reported that different artificial intelligence models showed correct response rates between 26%–38% in oral and maxillofacial surgery questions and had great difficulty answering questions that had visual components [22]. The study conducted by Kinikoglu in dentistry exams, also found that AI models had difficulty in questions with visual content [23]. Similar to this study, in our study, the success observed in knowledge-based and case-based questions could not be demonstrated in visual-based questions. While the DeepSeek R1 model failed in all questions with visual content, other models showed partial success. Given how widespread the use of visual diagnostic tools such as electrocardiogram, radiological images, and ultrasound are in emergency medicine practice, overcoming the limitations of LLMs in this area should be an important goal for future development.

One of the most important findings of our study is the difference in response consistency shown between the models. The Claude 3.5 model had the highest consistency (Fleiss’ Kappa = 0.68), while Gemini 2.0 had the lowest consistency (Fleiss’ Kappa = 0.49). When considering the use of artificial intelligence models in clinical settings, response consistency is critical to ensure reliability. A similar finding in the study by Lee et al. was that LLMs occasionally gave different answers to the same question, which can be an important issue for clinical reliability [1]. Moshirfar et al. also reported similar results [24]. In addition, response consistency is an indicator of how well models capture medical knowledge. A study by Yaneva et al. of USMLE sample items reported that the ability of artificial intelligence models to give consistent answers to repetitive questions reflects the depth of their medical knowledge [25]. In our study, the high consistency performance of the Claude 3.5 model suggests that this model is more stable in processing medical knowledge and producing consistent results.

In the Lee et al. study, artificial hallucination was defined as “LLMs producing inaccurate information as if it were true” and it was reported that 24.4% of the explanations of ChatGPT-4 and 47.2% of ChatGPT-3.5 were inaccurate or incorrect [1]. Zhu et al. also reported that ChatGPT can sometimes provide incomplete or inaccurate information when responding [5]. The study by Walters and Wilder reported that 55% and 18% of the short articles created with ChatGPT-3.5 and ChatGPT-4, respectively, contained fabricated references [26]. A study by Alkaissi and McFarlane on AI hallucinations highlighted that hallucinations can cause serious problems in the production of scientific content and that caution should be exercised in the clinical use of AI models [27]. In our study, inconsistent hallucinations were observed more frequently, particularly in the Gemini 2.0 and DeepSeek R1 models, whereas persistent hallucinations were observed less frequently in the ChatGPT-4o and Claude 3.5 models. These findings suggest that when evaluating the response reliability of AI models, response stability and hallucination tendencies should also be considered, not just overall accuracy rates.

Our study shows that the latest LLMs (ChatGPT-4o, Gemini 2.0, Claude 3.5, and DeepSeek R1) have made significant progress in addressing professional exam questions. However, our study has a number of limitations. First, as previous research suggests, the language of the questions can have a significant impact on the results. If questions are asked in Turkish and translated into English, discrepancies in meaning between the two languages may affect the accuracy of the answers. Second, because the sources of the questions (Tintinalli’s Emergency Medicine: A Comprehensive Study Guide, 9th Edition, and Rosen’s Emergency Medicine: Concepts and Clinical Practice, 10th Edition) were not included in the training data of the LLMs, this may have influenced their response accuracy. Finally, as the analyses of the Emergency Medicine YDUS results have not yet been published, the performances of ChatGPT-4o, Gemini 2.0, Claude 3.5, and DeepSeek R1 could not be compared with the actual exam outcomes.

As a result, among the four different artificial intelligence models used to answer the questions of the first-ever Emergency Medicine YDUS administered in Türkiye, the ChatGPT-4o model showed the best performance in terms of overall accuracy, while the Claude 3.5 model demonstrated the highest response consistency. The success rates of the models varied according to question types and difficulty levels. These results suggest that large language models can achieve high accuracy rates for knowledge and clinical reasoning questions in emergency medicine, but differ in terms of response consistency and hallucination tendency. While these models have significant potential for use in medical education and as clinical decision support systems, they need further development to provide reliable, up-to-date and accurate information. Future studies should more comprehensively evaluate the impact of large language models in medical education and their integration into clinical practice.

## Figures and Tables

**Table:** Comparison of overall and category-specific correct response rates of large language models.

		ChatGPT 4o n (%)	Gemini 2.0 n (%)	Claude 3.5 n (%)	DeepSeek R1 n (%)	p value*
**Overall accuracy**						
	Total (n = 60)	54 (90.0)	48 (80.0)	50 (83.3)	46 (76.7)	.065
	Visual (n = 2)	1 (50.0)	1 (50.0)	1 (50.0)	0 (0.0)	
	Knowledge (n = 26)	24 (92.3)	20 (76.9)	23 (88.5)	21 (80.8)	
	Case (n = 32)	29 (90.6)	27 (84.4)	26 (81.3)	25 (78.1)	
**Strict accuracy**						
	Total (n = 60)	57 (95.0)	55 (91.7)	53 (88.3)	51 (85)	.097
	Visual (n = 2)	1 (50.0)	1 (50.0)	2 (100.0)	0 (0.0)	
	Knowledge (n = 26)	25 (96.2)	23 (88.5)	23 (88.5)	23 (88.5)	
	Case (n = 32)	31 (96.9)	31 (96.9)	28 (87.5)	28 (87.5)	
**Ideal accuracy**						
	Total (n = 60)	58 (96.7)	57 (95.0)	56 (93.3)	55 (91.7)	.543
	Visual (n = 2)	1 (50.0)	1 (50.0)	2 (100.0)	0 (0.0)	
	Knowledge (n = 26)	25 (96.2)	25 (96.2)	25 (96.2)	25 (96.2)	
	Case (n = 32)	32 (100.0)	31 (96.9)	29 (90.6)	30 (93.8)	

* Statistical differences among the groups were analyzed using Cochran’s Q test.

Pairwise comparisons were conducted using McNemar’s test. For overall accuracy, a statistically significant difference was found between ChatGPT-4o and DeepSeek R1 (p = 0.021). Similarly, for strict accuracy, a statistically significant difference was observed between ChatGPT-4o and DeepSeek R1 (p = 0.031).

## Footnotes

1 2010-Intrance Examination for Subspecialty Training in Medicine (YDUS) [online] Website https://www.osym.gov.tr/TR,5149/2010-ydus-sonbahar-donemi-ek-yerlestirme-tercihlerin-alinmasi-27122010.html [accessed 10.03.2025]
